# Relationship Between Feed Olfactory Exploration and Consumption in Horses

**DOI:** 10.3390/ani16162599

**Published:** 2026-08-20

**Authors:** Elżbieta Wnuk, Wiktoria Janicka, Jarosław Łuszczyński, Angelika Wojtyniak, Wojciech Jagusiak, Anna Stachurska

**Affiliations:** 1 Department of Horse Breeding and Use, Faculty of Animal Sciences and Bioeconomy, University of Life Sciences in Lublin, 13 Akademicka Street, 20-950 Lublin, Poland; elzbieta.wnuk@up.edu.pl (E.W.); angelikawojtyniak2002@o2.pl (A.W.); anna.stachurska@up.edu.pl (A.S.); 2 Institute of Biological Basis of Animal Production, Faculty of Animal Sciences and Bioeconomy, University of Life Sciences in Lublin, 13 Akademicka Street, 20-950 Lublin, Poland; 3 Department of Genetics, Animal Breeding and Ethology, Faculty of Animal Science, University of Agriculture in Cracow, 21 Mickiewicz Avenue, 31-120 Cracow, Poland; jaroslaw.luszczynski@urk.edu.pl (J.Ł.); wojciech.jagusiak@urk.edu.pl (W.J.)

**Keywords:** olfaction, feeding behaviour, herbs, palatability, equids

## Abstract

The aim of the study was to evaluate the relationship between horses’ behaviour during olfactory exploration and following consumption of the same food containing herbs, either previously exposed to the herb odour or not. Twenty horses’ responses to 14 herbs were compared in a smelling test, when the horses sniffed a herb, and after 20 min a consumption test, when they consumed oats mixed with the same herb. Before the tests, the horses were exposed to the scent of seven herbs, whereas the remaining seven herbs were non-pre-exposed. The results show that horses consumed oats with herbal additives, regardless of whether pre-exposed, significantly longer than oats alone. The possibility of changes in feeding behaviour should be taken into account in horse feeding management when introducing new feeds or herbal supplements. Times of sniffing and consuming a herb were not correlated, whereas significant correlations in specific behaviours between the smelling and consumption tests were few and weak (from −0.162 to +0.146). It may be suggested that horses’ olfactory exploration of a feed has limited predictive value in relation to their subsequent consumption of it. The results should be treated as preliminary and considered a starting point for further research.

## Simple Summary

Smell and taste are important in food selection. The study aimed to evaluate the relationship between horses’ behaviour during olfactory exploration and following consumption of the same food. Twenty horses first sniffed 14 herbs and then consumed the herbs mixed with familiar oats. Each of the herbs was tested separately. Half of the herbs were olfactory pre-exposed and half non-pre-exposed to the horses. The results show that horses consumed oats with herbal additives significantly longer than oats alone, which may reflect horses’ caution toward herbal additives. Previous olfactory exposure to the plants did not influence further consumption. The possibility of changes in feeding behaviour should be taken into account in horse feeding management when introducing new feeds or herbal supplements. Times of sniffing and consuming a herb were not correlated, whereas correlations between specific behaviours accompanying the tests were few and weak. These outcomes may suggest that the information about the feed gathered by horses through smell is incomplete and must be supplemented by other senses. The results should be treated as preliminary and considered a starting point for further research.

## 1. Introduction

Horses have adapted to consume a varied plant-based diet by employing a patch-feeding strategy that allows them to carefully select desirable forage [1]. Diet selection and intake may be influenced by food availability, variety, appearance, organoleptic qualities such as taste, smell and texture [2,3], nutritional composition [4,5], as well as conditioned food preferences and aversions by which animals learn to associate the sensory properties of food with the metabolic consequences [6,7]. Van den Berg et al. [3] indicated that nutrient content appears to be the primary factor influencing dietary choices, but a non-nutritive sweet taste or an attractive odour may encourage feed intake in horses. In general, among herbivorous livestock species, the sensory properties of feed are usually perceived in the following order: appearance, aroma, texture, and flavour [8]. In horses, however, odour and taste seem to be more important than visual cues [9], while olfactory stimuli deliver the first information about a potential feed.

Horses have an acute sense of smell and may discriminate between different odours, showing preferences towards some scents [10]. The authors’ previous work showed that horses may recognise poisonous from non-poisonous plants based solely on olfactory cues [11]. In that study, horses explored edible plants longer and more intensively. In another study, we demonstrated that horses can be positively conditioned to detect scents at low intensities that are barely perceptible to humans [12]. Such a sensitive olfaction is crucial in many aspects of equines’ life, including feeding [10,13]. Vinassa et al. [14] observed that carrot, vanilla, milk protein with/without sugar, are preferred odours for ponies. Similar observations were made by Perry et al. [9] regarding anise aroma. It is crucial that horses, similar to other animals, smell an unknown odour of a diet before ingesting [15,16]. This is especially relevant given that poisonous plants are commonly found in rangelands and pastures worldwide [17].

The equine sense of taste is another aspect considered in the study. According to van den Berg et al. [3], for horses, taste is the secondary factor after nutritional value when selecting a diet, followed by smell. Francis et al. [18] tested oil-based palatants and observed increased palatability from anise and decreased palatability from orange flavours. In turn, ponies in the study of Khelil-Arfa et al. [19] accepted a broad range of flavours, showing a preference for apple-flavoured concentrate over raspberry. Horses can perceive four out of the five basic tastes, i.e., sweet, sour, salty, and bitter, while their ability to detect umami remains unknown [20]. Scant published research exists on the effect of sex and breed on taste preferences of horses. As shown by Janczarek et al. [21], taste preferences may vary between horses of different sexes and breeds. It is likely that prior exposure to specific odours early in life, access to pasture during youth, or general inter-individual variability may influence the food preferences of herbivores [22]. Studies conducted on other species of herbivorous farm animals, e.g., goats and cattle, also indicated differences in nutritional preferences and the botanical composition of the selected pasture sward depending on the age and sex of the animals [23,24]. It was found that differences between animal species also apply to humans, specifically, human perception of palatability differs from that of horses [25].

As prey species, horses are sensitive to different environmental stimuli, including, e.g., novel sounds and objects [26]. This caution is also evident in their reactions to unfamiliar foods and odours, a phenomenon known as neophobia [27]. Horses may exhibit a neophobic response to a novel odour or feed, which may diminish with subsequent exposures and consumption [3]. They tend to have a greater preference for familiar forages, regardless of energy intake [6], and familiar odours may increase the acceptance of a novel feed [7]. Christensen et al. [28] showed that an unknown smell may make horses more vigilant towards their surroundings. On the other hand, new odours increased feed palatability in ponies in a study by Vinassa et al. [14]. Therefore, neophobic responses to unfamiliar smells and tastes appear to be modulated by certain preferences. The senses of taste and smell are tightly connected, but studies on horses’ taste and olfactory sensitivity are also scarce [20]. However, as mentioned, it remains unknown whether horses can integrate odour and taste to perceive flavour as humans do [29]. From a biological perspective, it would be advantageous for horses to gather extensive information based solely on olfactory cues. In this way, they would be able to select the most attractive food source or avoid potential poisoning [11]. As we found in one of our previous studies [30], horses explore herbs known by smell and taste less intensively than those initially unfamiliar, but not less intensively than herbs known only by smell. Rørvang et al. [10] concluded that licking and biting behaviour during olfactory exploration may suggest that horses naïve to a taste of a substrate are able to link smell with taste. Perry et al. [9] noted that the horses’ first interaction with a feed’s new odour was sniffing rather than consumption. As noted by other authors [14,21], interest in a feed does not constitute evidence of its palatability.

Given the important roles of smell and taste in food selection and acceptance, and the limited knowledge in this field, the study aimed: (1) to investigate horses’ olfactory and feeding behaviour when presented with different herbal supplements, taking into account whether or not there has been prior intentional exposure to the scent of these plants and (2) to evaluate the relationship between horses’ behaviour during olfactory exploration of the herbs and during consumption of standard feed supplemented with these herbs. In this way, we wanted to assess whether horses’ olfactory behaviour toward the scent of a particular plant could predict their behaviour when consuming it, and hence whether the olfactory cue alone could provide horses with information about feed properties. The hypothesis of the study was that the addition of a herbal supplement affects horses’ olfactory and feeding behaviour. Specifically, prior intentional exposure to the scent of herbs is expected to shorten the duration of olfactory exploration and increase the willingness to consume it compared to feed with additives that have not been intentionally exposed beforehand. Moreover, the intensity of olfactory exploration of the plants is expected to be positively correlated with the intensity of consumption.

## 2. Materials and Methods

### 2.1. Horses

The study involved 20 adult horses owned by the University of Life Sciences in Lublin, Poland: 15 geldings (nine warmbloods and six ponies) and five mares (two warmbloods and three ponies) aged 7 to 23 (11.0 ± 4.3). It was not possible to include a balanced cohort of horses regarding sex and type. The horses were housed in the same facility for at least 4 years, with the majority of horses spending most of their lives there. Eight horses were born in the facility, six horses arrived ten to seventeen years before the experiment, and six joined four to eight years before. In the facility, the horses were housed in a stable-paddock system and kept in individual box stalls lined with triticale straw, with unlimited access to water and a salt lick. The horses were fed three times daily (at 6:00 AM, 1:00 PM and 8:00 PM), receiving meadow hay and oats with a vitamin and mineral supplement, in amounts individually tailored to their needs. They were turned out into ground paddocks with access to hay for 4 h per day in groups of a few individuals. At previous facilities in the same south-eastern Polish region, all horses had been kept in similar systems and at a young age they had access to pasture. The details of feeding and housing conditions in these facilities are not known and may have varied.

The mares were not in oestrus, pregnant or lactating during the experiment. The horses were under the daily care of an experienced handler, and one of the authors (EW) monitored their welfare. In addition, before and after the experiment, the horses underwent veterinary inspection, with particular attention to their general, respiratory and digestive health. All horses included in the study were clinically healthy, without signs of behavioural disorders, and had no respiratory or digestive problems or allergies. The horses were used for light leisure riding or hippotherapy for 1–2 h per day, six days per week, which, on average, corresponds to a moderate workload.

### 2.2. General Experimental Conditions

During the study, the horses were tested when sniffing and consuming various herbal plants with two different levels of olfactory familiarity, as determined by the experimental conditions (see Section 2.3 and Section 2.5.1 and Table 1). Depending on whether the horses were intentionally exposed to the scent of a given plant prior to the main study, the herbs were classified as pre-exposed (PE) or non-pre-exposed (NE). The horses initially smelled 50 g of a herb (smelling test, ST), and then consumed 5 g of the same herb mixed with 100 g of oats (consumption test, CT). In this manner, all herbs were tested individually. The herbal materials used and the detailed course of each stage of the experiment are described in the following subsections. All stages of the experiment, including the teaching phase, were conducted individually in each horse’s stall.

### 2.3. Herbs

The plant material used in the experiment consisted of 14 dried herbs that are not poisonous to horses and are sold as usual horse supplements (Equiart Czesława Grycz, Kruszyna, Poland). The herbs were purchased from a distributor in their original packaging, within their use-by dates, and once the packaging had been opened, the herbs were transferred to airtight plastic containers; each type of herb was kept separately. Herbal additives used in the study were not included in the tested horses’ diet as supplements. These herbal species are not found in a horse’s standard diet and contain potent, volatile chemical compounds; they are therefore suitable for studying how odours affect horses’ behaviour, and whether they trigger aversive reactions. They were selected randomly. We classified the herbs into seven PE and seven NE herbs (Table 1). Given that hay composition was not analysed, and considering the lack of detailed information about the horses’ previous feeding experiences in earlier facilities, we could not determine the studied horses’ level of familiarity with these plants and identify them as completely unfamiliar to the horses. Classifying the herbs into PE or NE, we were guided by common availability of the plants in the region where the horses lived, aiming to include the herbs possibly familiar into PE. None of the NE plants are species that grow naturally and widely in Polish meadows and pastures; the majority are plants of non-native origin, which in Poland are found in gardens or on farmland. The exceptions are a native tree (*Tilia cordata*) and a shrub (*Hippophae rhamnoides*), but these are not plants typical of meadow ecosystems. However, by exposing the horses to the scent of the PE herbs immediately before the study, we aimed to increase the degree to which they were familiar with the scent of a particular herb.

**Table 1:** The plant material used for the experiment—name of the species and type of material.

PE		NE	
*Calendula officinalis*	Flowers	*Nigella sativa*	Seeds
*Aronia melanocarpa*	Fruits	*Salvia officinalis*	Leaves
*Carum carvi*	Seeds	*Tilia cordata*	Flowers
*Rubus idaeus*	Leaves	*Ocimum basilicum*	Leaves
*Salvia rosmarinus*	Leaves	*Hippophae rhamnoides*	Fruits
*Rosa canina*	Fruits	*Vitex agnus-castus*	Fruits
*Trigonella foenum-graecum*	Seeds	*Foeniculum vulgare*	Seeds

PE—herbs pre-exposed to horses by smell. NE—herbs non-pre-exposed to horses.

### 2.4. Experimental Crib

**Figure 1:**
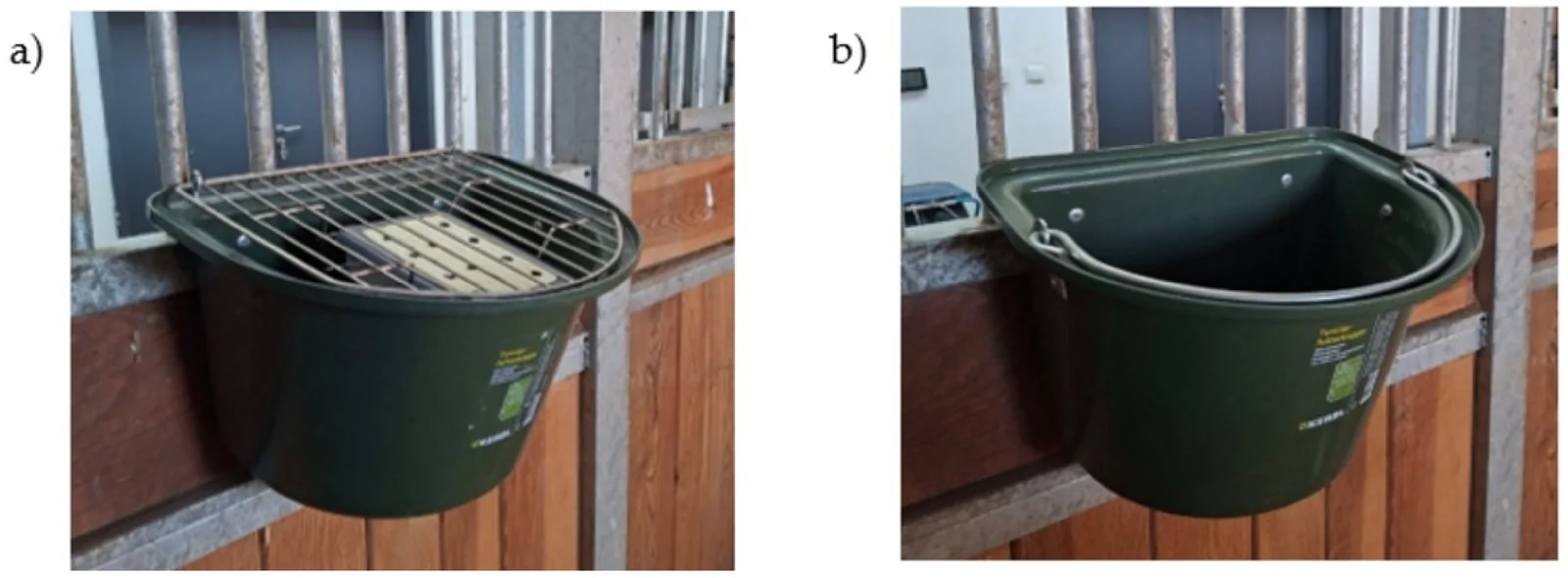
The ST crib (**a**) and CT crib (**b**) used in the experiment. ST—crib used in the smelling test, CT—crib used in the consumption test.

Two different experimental cribs, in addition to mangers from which the horses usually consumed concentrates, were used in the study. For the ST, the experimental crib (ST crib) was equipped with a mobile steel grate that prevented the contents from being eaten or observed by a horse, while allowing olfactory access. Directly beneath the steel grate, an opaque plastic box (20 cm long, 14 cm wide, and 5 cm high) was positioned. The lid of the box had 10 holes (1.2 cm in diameter) to release scent (Figure 1a). The crib was constructed according to the first model developed for the authors’ previous experiments and described in detail in previous papers [11,12,30]. During the control smelling test, two ST cribs were used, whereas during the main smelling test, one ST crib was used. For the CT, when the horses were consuming oats supplemented with herbal plants, another crib (CT crib; one during both control and main consumption test) was used that lacked both the mobile steel grate and the plastic box, thereby allowing the horses to access the feed (Figure 1b). The size and colour of the CT and ST cribs were the same. During the experiment, depending on the kind of test, an appropriate crib was hung on the front wall inside the horse’s stall at an approximate height of 120 cm for warmblood horses and 100 cm for ponies.

### 2.5. Study Design

#### 2.5.1. Teaching Phase

In the first stage, the horses were taught to approach and touch the empty ST crib with their mouth. When the horse was standing at least 1 m from the front wall of the stall, one of the experimenters entered the stall and hung the crib. After voluntarily approaching and touching the crib, the horse received a handful of oats (a feed familiar to the horses and readily consumed) from the experimenter. If a horse did not approach the crib on its own within 30 s, the experimenter led it to the crib. In this way, the horses’ interest in the crib was elicited, thereby enabling subsequent willingness to participate in the tests. The procedure was repeated daily for five consecutive days. After this time, a control trial was conducted to determine whether the horses met the criterion for the teaching phase. If a horse independently approached the crib within 10 s and touched it, it was considered to have successfully completed the conditioning process and was allowed to participate in subsequent stages of the study. Horses that did not fulfil the criterion would undergo the teaching phase again (5 days) and would then be retested. If they failed again, they would be excluded from the study. However, all the horses willingly approached the crib once it was hung after first five days of the teaching phase, which was considered a success at this stage.

In the second stage, the horses were intentionally exposed to the scent of PE herbs, to familiarise them with the scent of these herbs if novel, or to remind them and reinforce the association if they have encountered this scent before. Fifty grams of a herb were placed in the ST crib box, and the horse was led to it and allowed to smell it for 20 s. This quantity was chosen because this mass of dried plant material provides a large surface area from which essential oils are freely released, and in a sealed container this quantity allows a sufficient amount of aromas to accumulate in the space surrounding the dried material. If a horse abandoned the smelling after a few seconds, it was gently encouraged by holding its head close to the crib. Successive PEs were smelled at approximately 5 min intervals, one after another: four herbs in the morning (8:00–9:00 AM) and three herbs in the afternoon (5:00–6:00 PM), always in a different order. These activities were repeated for nine consecutive days, so that each PE was sniffed ten times to reinforce the horses’ familiarity with its scent. NE herbs were not used at all in this stage.

In the third stage, the horses were accustomed to eating from the CT crib. To achieve this, they were given a handful of oats, placed in the crib once daily for the following three days, prior to the main CT.

#### 2.5.2. Control Smelling Test

The goal of the control smelling test was to determine whether horses were sufficiently accustomed to the cribs, interested in them during exploration, and guided by scent rather than by objects to explore. This test was conducted by two experimenters (EW and WJ). Two identical ST cribs were hung on the front wall of the stall, 1 m apart, and the horses were allowed to explore them freely for 30 s. One crib was empty, and the other contained 100 g of oats. Half of the randomly selected horses had the crib with oats hung on the left side, and the other half on the right. One of the experimenters held the horse on a lead, with its head directed towards the front wall, positioned 1 m from the front wall (hereafter referred to as the starting position). After the other experimenter hung the crib, the horse was released, and the control measurement began. The experimenters measured the time spent exploring each crib (exploration time), the number of approaches to each crib (exploration frequency; Table 2), and recorded the order in which a horse explored the cribs. It was assumed that a longer time of exploration, more approaches, and first approaching the crib with oats would indicate that the horse perceived the smell of oats in the filled crib and was not only interested in the crib as an object to play with. The control smelling test was performed once, one day before the beginning of the main tests, starting 2 h after midday feeding (3:00 PM).

#### 2.5.3. Control Consumption Test

The control oats consumption test was conducted to establish a standard consumption time and feeding behaviour for each horse. In addition, the results of this control test were compared with those of the main consumption test to determine whether the herbs added to oats affected the parameters studied. The CT crib with 100 g of oats was used. The horses were released from the starting position, similar to the control smelling test. Experimenters measured the consumption time and the number of breaks, and recorded all behaviours observed as during the main consumption test (Table 2). The control consumption test was performed once, on the day before the main tests began, just after the control smelling test (about 30 min later; beginning around 3:30 PM). The horses were tested in the same order as in the control smelling test.

#### 2.5.4. Main Testing

The main testing was conducted on consecutive days, from Monday to Friday, between 3:00 PM and 5:00 PM. The horses had already eaten their midday meal, and it was still 3 h to the evening feeding. The main testing was conducted by two experimenters (EW and WJ). Over 14 consecutive days, the horses were given one herb to explore and consume—a different herb on each day. This resulted in a total of 14 research days per horse. The horses were presented with PE and NE herbs in alternating order. The order of the herbs within the PE and NE groups was random on each day. Half of the horses were tested using one plant order (starting with PE on the first day), and the remaining individuals were tested in reverse order of the horses’ stall positions (starting with NE on the first day).

- ST—The horses had the opportunity to smell one of the herbs for 30 s. One of the experimenters held the horse on a lead in the stall in the starting position (as in the phases of the control smelling test and the control consumption test), while the second experimenter simultaneously hung the ST crib. The first experimenter then released the horse. They both immediately left the stall, and the test began when the horse approached the crib and touched it with its nostrils.
- CT—After 20 min, one of the experimenters entered the stall and brought the horse back to the starting position. The other experimenter then hung the CT crib filled with 100 g of oats mixed with 5 g of the same herb that had been previously smelled (oats+PE or oats+NE). The horse was released, the experimenters immediately left the stall, and the test began when the horse put its head into the crib. Based on the control consumption test, the maximum CT consumption time was assumed to be 180 s. If the horse did not eat the feed within this time, the consumption time was stopped, and the leftovers were collected and weighed.

#### 2.5.5. Variables

During control and main tests, behaviours determined in the time unit were recorded: the time [s] and frequency [number of events] as well as the occurrence [0–1 sampling method] [31] of different behavioural characteristics were assessed, in accordance with the prepared ethogram (Table 2). The horses’ behaviour was assessed live by two experimenters (EW, WJ), both highly experienced in assessing horse behaviour. One of the experimenters noted behaviours using a 0–1 sampling method (EW), and the second experimenter (WJ) recorded behaviours in frequency and time units. Both of them were blind to the herb species added to the crib; they were given a ready-to-use crib containing a herb, which had been prepared by an assistant in another room. Prior to the control and main tests, inter-observer percentage agreement was assessed using 10 30 s olfactory and consumption trials with oats (as in the ST and CT). Each experimenter recorded both behaviours assessed with a 0–1 sampling method and those determined in the time unit. The average percentage agreement between observers was calculated based on the percentage agreement for each rated variable across both behaviour categories; for behaviours in the 0–1 category, this was calculated as the number of consistent ratings/the total number of ratings * 100%, and for behaviours in the time category as the lower value assigned by one experimenter/the higher value assigned by the other observer * 100%. For behaviours in the 0–1 category, agreement was 98%, and for behaviours in the time category, 92%. For this reason, i.e., such a high agreement, to facilitate live observation, the observed categories were divided between the two observers.

### 2.6. Statistical Analysis of Data

The data were analysed in three groups. The first group comprised two variables recorded during the teaching phase: exploration time and exploration frequency. The second group included four traits related to the ST: exploration time, exploration frequency, crib nibbling, and crib licking. The third group consisted of traits recorded during the CT: consumption time, number of breaks, drooling, crib licking, chewing outside the crib, and the amount of leftover food.

For the discrete variables analysed in this study, the degree of dispersion was evaluated using the SAS GENMOD procedure. No overdispersion was found, and the Poisson distribution was applied. Count outcomes were subsequently analysed using generalised linear mixed models (GLMMs) with a Poisson distribution.

The ‘exploration time’ and ‘consumption time’ variables were tested for normality. Due to skewness, they were log-transformed (using the natural logarithm), after which the distribution did not differ significantly from normal (SAS UNIVARIATE procedure). These variables were then analysed using the SAS MIXED procedure, keeping a linear model consistent with the one used for categorical variables, but under the assumption of normality.

A random effect of horse was included in all models to account for within-subject correlation, since each animal underwent a series of taste and olfactory tests using various herbs. Fixed effects varied depending on the trait group. For traits in the first group, only the effect of the crib (empty/oats) was included. For traits in the second and third groups, the fixed effects of horse sex, horse type, and herb type (PE, NE), as well as the interactions between horse sex and herb type and between horse type and herb type, were included. Significant effects of herb type and interactions were further examined by comparing least squares means across levels using the Tukey–Kramer adjustment.

To evaluate within-subject interactions while avoiding pseudo-replication, count metrics were transformed using the Freeman–Tukey transformation (√Y + √(Y + 1)), whereas ‘exploration time’ and ‘consumption time’ were log-transformed. Subsequently, all variables were analysed using a linear mixed model (SAS, PROC MIXED). The model included the fixed interaction effect of herb type and variable (herb_type x variable). To account for repeated measures, herb × horse was specified as the random subject unit using an unstructured covariance matrix (TYPE = UN) to model the correlations among variables. Finally, model-conditioned within-subject residuals were extracted to calculate pairwise correlations between variables and their corresponding *p*-values using Pearson correlation with Fisher’s z-transformation (SAS, PROC CORR).

All analyses were performed using SAS, 9.4 version (SAS Institute Inc., Cary, NC, USA).

## 3. Results

### 3.1. Smelling Tests

During the smelling control test, the horses approached the crib containing oats first in 70.0% of cases. They also explored it for almost five times longer (*p* < 0.001) and almost twice as frequently (*p* = 0.021) than the empty crib (Table 3). Individual results indicated that all horses passed the control smelling test.

When comparing the olfactory exploration of PE and NE herbs by horses, it was found that horses explored NE and PE for a similar time (*p* > 0.05; Table 4). No significant differences were observed in the frequency of exploration or in the occurrence of crib nibbling and crib licking, either.

Considering the impact of horses’ biological traits, no significant differences were found between mares and geldings, nor between warmblood horses and ponies, in olfactory exploration of PE and NE. However, within-group comparisons showed that geldings explored NE longer than PE (*p* = 0.037; Table 5).

### 3.2. Consumption Tests

When comparing the control consumption of oats, oats+PE, and oats+NE, only consumption time differed significantly. It was found that horses ate oats alone for a significantly shorter time than oats+PE (*p* < 0.001) or oats+NE (*p* < 0.001; Table 6). No significant differences were found between oats+PE and oats+NE.

Horse sex and type did not significantly differentiate the feeding behaviour. Taking into account within-group comparisons, it was noted that geldings consumed oats alone for a significantly shorter time than oats+PE (*p* = 0.004) and oats+NE (*p* = 0.002), but no differences were found in the consumption time of oats+PE and oats+NE (Table 7). Similar observations were made for ponies (for oats compared to oats+PE and oats+NE; *p* = 0.004, *p* = 0.005, respectively). In the case of warmbloods, a significant difference was found only between the consumption time of oats and oats+NE (*p* = 0.028).

### 3.3. Correlations Between Variables Recorded in ST and CT

The correlation between the exploration time during the smelling test and the consumption time amounted to −0.056 (*p* = 0.347), hence it can be inferred that the times were not correlated. Due to statistical considerations, continuous quantitative variables could not be directly compared with count data of specific behaviours, hence the correlation was not included in Table 8. Comparing behavioural count metrics (Table 8), three significant, although weak correlations between the ST and CT tests were found.

## 4. Discussion

The sense of smell plays a crucial role in various behavioural contexts in the lives of horses, including dietary habits [3,11,30,32,33,34]. Touch and taste follow smell in the sensory hierarchy. These senses are active during feeding and may function in opposite ways: for example, a scent might attract a horse, whereas the texture or taste of a plant could discourage consumption [14,21,35,36]. The current study examined the relationship between smell and taste in horses. The research involved assessing horses’ olfactory and feeding behaviours as they smelled and consumed 14 different herbs, some of which the horses had been intentionally exposed to previously. The study also considered the influence of biological traits, such as sex and horse type.

The fact that, during the control smelling test, the crib filled with oats was chosen more readily and explored more willingly than the empty crib clearly indicated that the horses studied were guided in this task by the known oats’ scent rather than by a general willingness to play or explore the object. Thus, food that is well known for its smell and taste, and likely for the positive associations linked to its metabolic effects (e.g., providing energy, being free from toxic compounds) [3,4] is easily detected on the basis of olfactory cues alone. However, the horses’ prior exposure to herb scents in the PE group did not affect how they explored scents compared to the NE group, which had not been intentionally exposed before the study. This result contradicts our assumption that a less familiar scent would be associated with more intense exploration, whereas a previously encountered scent would be explored less intensively due to rapid recognition. The assumption was based on the findings of our previous study, in which horses explored herbs they knew by smell and taste less intensively than herbs that were initially unfamiliar to them [30]. The difference may result from the fact that familiarisation with a food through multiple senses: smell, touch, and taste, provides horses with more information about its properties, allowing for quicker recognition in the future [3,7]. Therefore, its olfactory exploration during subsequent encounters is shorter. Moreover, in this study, all tested plants were edible for horses and could therefore have been perceived as neutral or more or less attractive. In contrast, in our other study, horses exposed to the scent of unfamiliar poisonous and non-poisonous plants spent more time sniffing the non-poisonous plants, likely perceiving them as more attractive [11]. These findings suggest that the intensity of olfactory exploration varies with context. Rørvang et al. [10] previously showed that presenting new odours increased sniffing duration in horses. Similar observations have been made in cattle [37]. However, prolonged sniffing of novel odours may also reflect greater interest. Rørvang et al. [10] also found that sniffing duration was affected by specific odours, with one odour eliciting longer sniffing times than others. Preferences for certain plant scents were also noted in one of our previous studies [11].

The CT results indicate that oats are consumed more quickly when fed alone than when fed with herbal additions, regardless of the herbs’ familiarity. This observation suggests a preference for well-known forages and is consistent with findings reported by van den Berg et al. [6]. It may support the hypothesis on food neophobia in horses, indicating a tendency to be cautious toward novel feeds [3,6,38]. Although it cannot be ruled out that the horses may have been exposed early in life to the herbs included in the study through hay or in previous housing environments, herbal additions used in the study were not as familiar to the horses as their regular feed (oats). The addition of herbal supplements may have influenced their perception of the standard feed [39]. The possibility of changes in feeding behaviour should be taken into account in animal feeding management, not only when introducing new feeds, but also during periodic dietary changes or when using herbal supplements [40].

In the current study, no significant differences in consumption time were observed between NE and PE plants, suggesting that prior odour exposure to PE plants did not sufficiently influence feeding behaviour during initial consumption. The information horses gathered on the basis of increased familiarity with a herb was not adequate to consume the herb without caution. This fact, together with the above-mentioned finding on the quicker consumption of oats without herbal additives, may discourage horse owners from the rapid introduction of supplements into their horses’ diets. The potential causes of the lack of significant difference in the consumption time between PE and NE may be similar to those underlying the lack of differences in their olfactory exploration, namely the absence of multisensory exposure to PE plants prior to the study [3,7], the absence of negative experiences associated with the consumption of these plants in the past [3,4], as well as the random assignment of herbs to the PE and NE groups, without accounting for their potentially varying attractiveness to horses [10,11,19].

The sex and type (warmblood, pony) of the horses were irrelevant to the variables studied: no differences between mares and geldings or between warmbloods and ponies were observed in either ST or CT. The authors made similar observations in previous studies [11,30]. On the other hand, Janczarek et al. [21] found that the taste preferences of horses depend on their sex and breed. However, when differences within individual groups of horses were considered in the present study, geldings appeared to be more responsive to the herbal additions compared to mares. Unlike them, the geldings consumed the mixtures of oats and herbs (both PE and NE) longer than oats alone. They also explored NE herbs significantly longer than PE. This result may suggest that geldings are more olfactory-sensitive than mares, in accordance with our previous observations [30], and were more cautious during consumption of standard feed with herbal additives. However, the results of this study regarding sex differences should be interpreted with caution due to the large imbalance between the groups, and they may serve rather as a starting point for further research into differences in olfactory exploration and feeding behaviours between mares and geldings, or even more interestingly, stallions.

Considering the horse type, both ponies and warmbloods consumed oats alone quicker than oats+NE. Ponies consumed oats with an addition of PE significantly longer than oats alone. These results may indicate that cautious behaviour towards the addition of herbal supplements into a standard feed is more pronounced in ponies (applies to both PE and NE) than warmbloods. It is difficult to interpret these findings, which should be subjected to more comprehensive studies. Currently, they are based only on the consumption time variable. It is probable that ponies included in the study (Felin pony breed), as more primitive horses [41], may be characterised by a stronger expression of instincts important for survival. Interestingly, in our previous study we also found that warmbloods seemed more cautious when exposed to the scent of less familiar plants [30]. However, it should be noted that horses may have individual preferences for certain flavours and may refuse to eat a feed they dislike [19,42]. Thus, the differences in responses between the two groups may have resulted from differences in their preferences for the herbs used in the study, their prior experience with these plants, or the properties of the herbs themselves, as previously mentioned. This possibility should be considered when interpreting the results of this study.

There is some evidence that horses may link odours and tastes, but studies on horses’ mutual sensitivity to tastes and scents are scarce [18,25]. The present study showed that the most important variables, i.e., exploration time [11,30] and consumption time, are not correlated. Statistically, the quantitative variables could not have been compared to other variables with a non-parametric distribution. Correlations between ST and CT of the latter variables were few and weak which is consistent with the lack of correlation shown by the time variables. The result suggests that when consuming, the horses were not directed by the previous smelling of a herb which contradicts the second part of our hypothesis. Future studies should account whether the prior smelling should be longer and whether the interval before consuming should be shorter. However, it can be indicated that the link between the olfactory exploration and the following consumption is not observed. Previously, Francis et al. [18] observed a moderate positive correlation between the first product smelled and the first product consumed. Regarding the specific behaviours, the more frequent crib licking in ST corresponded to more frequent crib licking in CT, and less frequent chewing outside a crib as well as more frequent exploration in ST corresponded to crib licking in CT. Our previous study [11] demonstrated that licking the crib indicated interest in its contents, as the recognition of a positively conditioned odour was associated with this behaviour. In the study by Stachurska et al. [39] on horse feeding behaviour, dry feed led to more frequent breaks in intake (associated with chewing outside the crib) than wet or sweetened feed, indicating a lower willingness to consume it. Hence, it seems that in the present study, licking the crib could be a sign of liking the feed or a need to explore it and making wet, whereas chewing outside the crib could reflect reduced interest in the feed, especially given the fact that horses did not extend their heads beyond the crib and did not leave any leftovers during the control trial (well-known feed; oats). These observations may suggest that, to some degree, an interest in an odour may be associated with perceiving the feed as more attractive, and hence that horses can gather some information about feed properties based on olfactory cues alone. However, as mentioned above, although the correlations in specific behaviours between ST and CT were statistically significant, they were weak and rare. Therefore, the results of our study should be treated rather as preliminary and considered a starting point for further research on the relationship between sniffing and feed intake, which may be important for increasing acceptance of new feeds in horses [9,14]. It should be noted that sniffing behaviour does not necessarily correspond to feeding behaviour [14,21]. For example, Vinassa et al. [14] concluded that a preference for a novel odour does not necessarily lead to increased intake. Thus, a thorough analysis of both olfactory and feeding behaviours, along with their potential underlying causes, is required. It is possible that olfactory information alone is incomplete for horses and must be supplemented with taste and perhaps also tactile sensations. A more thorough understanding of how horses perceive feed is particularly important at present, when horses are often fed complete industrial feeds composed of many ingredients and supplemented with additives, which can create a mixture of flavours and odours.

The current study addressed an important topic regarding horse olfactory and taste senses, but it has certain limitations. It focused on horses’ olfactory and feeding responses to a mixture of oats and different herbs and was based on behavioural assessment. The authors are aware that it is not possible to determine with certainty what exactly may have caused a given behaviour, as horses’ responses could have been influenced by many factors, ranging from individual preferences and the animal’s physiological state on a given day, to the varying composition of different components in the herbs, as well as differences in the intensity of their aroma. The horses studied were differentiated by the origin facility; hence, their feeding and housing conditions, including familiarity with different plants encountered in pastures when they were young, could have varied. Therefore, we cannot rule out the possibility that the studied horses had come into contact with the plants used in the study earlier, before reaching the current facility, which may have affected the results. Further, because the sex ratio in the material was unbalanced, the conclusions about sex differences in response to the studied herbs should be treated with caution The selection of herbs was random and their assignment to PE and NE groups followed assumptions based only on environmental occurrence. It could not be determined whether the plants in a given group were inherently more attractive to horses. We tried to control this factor by ensuring that each group contained different plant parts, such as seeds, leaves, or fruits. However, differences in smell intensity or palatability of the herbs were not checked prior to the study. In the present study, we did not perform an analysis comparing the horses’ responses to the 14 herbs, as this would require adding another factor to a model that already included three fixed effects (sex, horse type, and herb type) for a study group comprising only 20 individuals. Furthermore, without accounting for the content of various chemical constituents in the specific herbs, drawing conclusions regarding the biological mechanisms underlying the olfactory and feeding behaviours of the horses would still be difficult. Therefore, when interpreting the results, it must be taken into account that they may have been influenced by individual preferences or plant-specific differences (e.g., smell and taste intensity and nutrient content), as well as the division of plants into PE and NE. Future studies should account for the effects of each individual herb in the analysis, which would help determine whether prior exposure to a scent is important or whether horses’ responses depend more on the specific herb. In addition, it remains unclear whether the number and duration of exposures to the odours of PE plants in the teaching phase were sufficient to justify their classification as PE plants. This aspect should be considered in further studies. We also cannot exclude the possibility that the adopted order of exposure to the herbs, although divided between two groups of horses, may have influenced the study results because the horses became accustomed to the experimental procedure. Finally, the attractiveness of a given feed is also influenced by different characteristics, such as texture and nutrient content. Neither in the present study nor in any of the authors’ previous studies [11,12,30] were these factors included in the analysis. Conducting an analysis that integrates these factors into an assessment of olfactory exploration and feeding behaviour in horses would be advantageous.

Summing up, it was not expected that the study would provide comprehensive answers on the relationship between olfactory exploration and feed consumption in horses, but rather to raise the issue. The current findings should be extensively studied in future, possibly avoiding at least some of the indicated limitations. Understanding how horses use olfaction to select food and how olfaction influences consumption is of utmost importance for feed management, horse health and welfare, e.g., in preventing the ingestion of unsuitable plants, enriching feeding environments and developing supplementation strategies that regard horses’ preferences.

## 5. Conclusions

The addition of herbal supplements to the standard feed was associated with increased consumption time. The possibility of changes in feeding behaviour should be taken into account in horse feeding management when introducing new feeds or herbal supplements. Prior intentional exposure to the scent of herbs did not affect changes in olfactory exploration or feeding behaviour toward these plants. It may be suggested that olfactory information alone is incomplete for horses and must be supplemented with taste and perhaps also tactile sensations. The olfactory exploration time and the consumption time of the same feed measured after 20 min were not correlated. Correlations between olfactory exploration and the following consumption in specific behaviours were few and weak (crib licking, chewing outside the crib) and poorly indicated an effect of olfactory cues on the feeding behaviour. The results should be treated as preliminary and considered a starting point for further research. Geldings seem to be more olfactory sensitive, and both ponies and geldings seem more cautious toward herbal additions than mares and warmbloods, respectively. However, the imbalance between the groups in this study warrants cautious interpretation of the results. A thorough understanding of how horses perceive feed is important for feed management, horse health and welfare.

## Figures and Tables

**Table 2:** Variables recorded during testing the horses.

Variable	Unit of Measure	Description
Control smelling test and smelling test (ST)		
Exploration time	s	Total time spent on exploration-related events, i.e., any interaction with the crib, such as touching, sniffing, licking, nibbling; measured with an accuracy of 1 s
Exploration frequency	number per 30 s	The total number of consecutive events during which the horse explored the crib; events counted separately when separated by an interval of at least 3 s
Crib nibbling *	0–1 sampling method **	The horse’s jaws were closed, and the upper lip was moved upward and downward against the crib with curiosity and lack of fear
Crib licking *	0–1 sampling method **	The horse stuck out its tongue, contacting the crib
Control consumption test and consumption test (CT)		
Consumption time	s	Total time spent on consuming the experimental sample of oats with herbs measured with an accuracy of 1 s
Number of breaks	number of events	Number of breaks in feed consumption lasting at least 10 s
Crib licking	0–1 sampling method **	The horse stuck out its tongue, contacting the crib
Drooling	0–1 sampling method **	The horse produced a significant amount of saliva, which was clearly visible outside its mouth
Chewing outside the crib	0–1 sampling method **	The horse’s head was clearly outside the crib while chewing a mouthful of feed for at least 10 s
Spitting out ***	0–1 sampling method **	The horse spat feed from its mouth or pushed it out with its tongue
Lip licking ***	0–1 sampling method **	The horse licked its lips, clearly sticking out its tongue
Amount of leftovers	g	Total mass of feed leftovers not eaten by the horse, weighed with an accuracy of 1 g

* not recorded in control smelling test. ** non-occurrence/occurrence during 30 s. *** the behaviour did not occur or was extremely rare, hence, it was not taken into account in the final analysis.

**Table 3:** Comparison of time [s] and frequency [number per 30 s] of exploration by horses of an empty crib vs. a crib containing oats in the control smelling test.

	*n*	Empty Crib		Oats Crib	
		Mean	SD	Mean	SD
Exploration time	20	3.2 ^A^	2.55	15.1 ^A^	6.43
Exploration frequency	20	1.3 ^a^	0.91	2.3 ^a^	0.98

*n*—number of observations. Difference between means in rows marked with the same capitals (A) is statistically significant at *p* < 0.01. Difference between means in rows marked with the same small letter (a) is statistically significant at *p* < 0.05.

**Table 4:** General comparison of olfactory exploration of pre-exposed and non-pre-exposed herbs by horses in the smelling test.

	*n*	PE		NE	
		Mean	SD	Mean	SD
Exploration time	20	9.5	7.35	11.3	7.55
Exploration frequency	20	1.8	0.87	1.9	0.89
Crib nibbling	20	0.2	0.57	0.3	0.59
Crib licking	20	0.1	0.35	0.1	0.34

*n*—number of observations. PE—herbs pre-exposed to horses by smell. NE—herbs non-pre-exposed to horses.

**Table 5:** Comparison of olfactory exploration of pre-exposed and non-pre-exposed herbs in relation to horse sex and horse type in the smelling test.

	PE		NE	
	Sex of horse			
	Mare *n* = 5	Gelding *n* = 15	Mare *n* = 5	Gelding *n* = 15
Exploration time	10.8 ± 7.55	9.1 ^a^ ± 7.27	10.8 ± 8.43	11.4 ^a^ ± 7.27
Exploration frequency	1.9 ± 0.94	1.7 ± 0.84	1.9 ± 0.94	2.0 ± 0.88
Crib nibbling	0.3 ± 0.62	0.2 ± 0.55	0.3 ± 0.56	0.3 ± 0.60
Crib licking	0.2 ± 0.38	0.1 ± 0.34	0.1 ± 0.37	0.1 ± 0.33
	Type of horse			
	Warmblood *n* = 11	Pony *n* = 9	Warmblood *n* = 11	Pony *n* = 9
Exploration time	8.7 ± 7.28	10.5 ± 7.37	11.7 ± 7.80	10.8 ± 7.27
Exploration frequency	1.6 ± 0.73	2.0 ± 0.97	1.9 ± 0.89	2.0 ± 0.89
Crib nibbling	0.1 ± 0.33	0.3 ± 0.74	0.2 ± 0.48	0.3 ± 0.70
Crib licking	0.1 ± 0.33	0.2 ± 0.37	0.1 ± 0.33	0.1 ± 0.35

*n*—number of observations. PE—herbs pre-exposed to horses by smell. NE—herbs non-pre-exposed to horses. Difference between means in rows marked with the same small letter (a) is statistically significant at *p* < 0.05.

**Table 6:** General comparison of feeding behaviours during consumption of oats vs. the mixture of oats and pre-exposed and non-pre-exposed herbs by horses.

	*n*	Oats (Control Consumption Test)		Oats+PE (Consumption Test)		Oats+NE (Consumption Test)	
		Mean	SD	Mean	SD	Mean	SD
Consumption time	20	82.9 ^AB^	19.05	113.5 ^A^	0.50	117.4 ^B^	36.60
Number of breaks	20	0.0	0.00	0.1	0.27	0.2	0.41
Drooling	20	0.1	0.22	0.2	0.37	0.1	0.27
Crib licking	20	0.1	0.27	0.6	0.50	0.5	0.50
Chewing outside a crib	20	0.0	0.00	0.5	0.88	0.6	0.86
Leftovers	20	0.0	0.00	0.7	3.61	3.9	12.09

*n*—number of observations. PE—herbs pre-exposed to horses by smell. NE—herbs non-pre-exposed to horses. Oats+PE—oats mixed with herbs pre-exposed to horses by smell. Oats+NE—oats mixed with herbs non-pre-exposed to horses. Differences between means in rows marked with the same capitals (A, B) are statistically significant at *p* < 0.01.

**Table 7:** Comparison of feeding behaviours during consumption of oats vs. the mixture of oats and pre-exposed and non-pre-exposed herbs in relation to horse sex and horse type.

	Oats (Control Consumption Test)		Oats+PE (Consumption Test)		Oats+NE (Consumption Test)	
	Sex of horse					
	Mare *n* = 5	Gelding *n* = 15	Mare *n* = 5	Gelding *n* = 15	Mare *n* = 5	Gelding *n* = 15
Consumption time	85.9 ± 16.24	81.4 ^AB^ ± 20.89	111.7 ± 34.23	114.4 ^A^ ± 33.13	114.2 ± 32.92	119.1 ^B^ ± 38.39
Number of breaks	0.0 ± 0.00	0.0 ± 0.00	0.1 ± 0.24	0.1 ± 0.28	0.1 ± 0.34	0.2 ± 0.45
Drooling	0.0 ± 0.00	0.1 ± 0.28	0.1 ± 0.31	0.2 ± 0.40	0.1 ± 0.25	0.1 ± 0.28
Crib licking	0.7 ± 0.49	0.8 ± 0.44	0.6 ± 0.50	0.5 ± 0.50	0.5 ± 0.50	0.5 ± 0.50
Chewing outside a crib	0.0 ± 0.00	0.0 ± 0.00	0.4 ± 0.67	0.6 ± 0.97	0.7 ± 0.88	0.6 ± 0.85
Leftovers	0.0 ± 0.00	0.0 ± 0.00	0.5 ± 2.41	0.9 ± 4.12	2.0 ± 7.87	4.8 ± 13.68
	Type of horse					
	Warmblood *n* = 11	Pony *n* = 9	Warmblood *n* = 11	Pony *n* = 9	Warmblood *n* = 11	Pony *n* = 9
Consumption time	82.2 ^a^ ± 21.22	83.9 ^CD^ ± 16.61	109.6 ± 33.63	119.3 ^C^ ± 32.53	116.1 ^a^ ± 36.99	119.5 ^D^ ± 36.99
Number of breaks	0.0 ± 0.00	0.0 ± 0.00	0.1 ± 0.26	0.1 ± 0.29	0.2 ± 0.40	0.2 ± 0.44
Drooling	0.0 ± 0.00	0.1 ± 0.35	0.2 ± 0.40	0.1 ± 0.31	0.1 ± 0.30	0.1 ± 0.23
Crib licking	0.8 ± 0.45	0.0 ± 0.46	0.6 ± 0.50	0.6 ± 0.50	0.6 ± 0.50	0.4 ± 0.49
Chewing outside a crib	0.0 ± 0.00	0.0 ± 0.00	0.4 ± 0.63	0.6 ± 1.16	0.6 ± 0.92	0.6 ± 0.78
Leftovers	0.0 ± 0.00	0.0 ± 0.00	0.6 ± 2.94	0.9 ± 4.46	3.7 ± 11.48	4.1 ± 13.04

*n*—number of observations. PE—herbs pre-exposed to horses by smell. NE—herbs non-pre-exposed to horses. Oats+PE—oats mixed with herbs pre-exposed to horses by smell. Oats+NE—oats mixed with herbs non-pre-exposed to horses. Differences between means in rows marked with the same capitals (A, B, C, D) are statistically significant at *p* < 0.01. Difference between means in rows marked with the same small letter (a) is statistically significant at *p* < 0.05.

**Table 8:** Correlations between variables recorded in Smelling Test and Consumption Test.

	Number of Breaks CT	Crib Licking CT	Drooling CT	Chewing Outside the Crib CT	Amount of Leftovers CT
Exploration frequency ST	−0.035 *p* = 0.560	0.123 *p* = 0.040	−0.055 *p* = 0.360	0.037 *p* = 0.541	−0.056 *p* = 0.346
Crib nibbling ST	−0.078 *p* = 0.193	0.077 *p* = 0.200	−0.042 *p* = 0.479	−0.071 *p* = 0.238	−0.102 *p* = 0.090
Crib licking ST	−0.104 *p* = 0.083	0.146 *p* = 0.014	−0.065 *p* = 0.277	−0.162 *p* = 0.007	−0.107 *p* = 0.073

ST—smelling test. CT—consumption test.

## Data Availability

The data presented in this study are available on request from the corresponding author.

## Abbreviations

The following abbreviations are used in this manuscript:

PE	herbs pre-exposed to horses by smell
NE	herbs non-pre-exposed to horses
ST	smelling test
CT	consumption test
